# The Impact of Cardiac Lipotoxicity on Cardiac Function and Mirnas Signature in Obese and Non-Obese Rats with Myocardial Infarction

**DOI:** 10.1038/s41598-018-36914-y

**Published:** 2019-01-24

**Authors:** Gema Marín-Royo, Adriana Ortega-Hernández, Ernesto Martínez-Martínez, Raquel Jurado-López, María Luaces, Fabián Islas, Dulcenombre Gómez-Garre, Beatriz Delgado-Valero, Esther Lagunas, Bunty Ramchandani, Mónica García-Bouza, María Luisa Nieto, Victoria Cachofeiro

**Affiliations:** 1 Departamento de Fisiología, Facultad de Medicina, Universidad Complutense de Madrid and Instituto de Investigación Sanitaria Gregorio Marañón (IiSGM), Madrid, Spain; 2 Laboratorio en Biología Vascular, Hospital Clínico San Carlos-IdISSC, Madrid, Spain; 3 Ciber de Enfermedades Cardiovasculares (CIBERCV), Instituto de Salud Carlos III, Madrid, Spain; 4 Servicio de Cardiología, Instituto Cardiovascular, Hospital Clínico San Carlos, Madrid, Spain; 5 Departamento de Cirugía Cardiaca, Hospital Clínico San Carlos, Madrid, Spain; 6 Departamento de Cirugía Cardiaca, Hospital Universitario de Canarias, Tenerife, Spain; 7 Instituto de Biología y Genética Molecular, CSIC-Universidad de Valladolid, Valladolid, Spain

## Abstract

Cardiac lipotoxicity is involved in the cardiac functional consequences associated with obesity. Therefore, the aim of this study was to explore whether changes in the mitochondrial lipid cardiac profile could reflect differences in cardiac function and structure in obese and non-obese rats with myocardial infarction (MI). Whether these changes can also be reflected in a specific plasma miRNA signature as markers of cardiac damage was also evaluated. Rats were fed with either standard (3.5% fat) or high fat diet (35% fat) for 6 weeks before the induction of MI and sacrificed 4 weeks later. MI showed cardiac lipotoxicity independently of the presence of obesity, although obese and non-obese rats did not present the same cardiac lipid profile at mitochondrial level. Several cardiac lipid species in mitochondria, including cardiolipins and triglycerides, were associated with myocardial fibrosis, with mitochondrial triglyceride levels being independently associated with it; this supports that lipotoxicity can affect cardiac function. MI down-regulated plasma levels of miRNA 15b-5p and 194-5p in obese and non-obese animals, which were associated with cardiac function, mitochondrial lipids and myocardial fibrosis, with miRNA 15b-5p levels being independently associated with cardiac fibrosis. This could support that lipotoxicity could affect heart function by modulating plasma miRNAs.

## Introduction

Obesity is associated with a higher risk of cardiovascular morbidity and mortality, which has been explained through the impact of low-grade chronic inflammation, increased oxidative stress and the associated cardiometabolic alterations^1–3^. Cardiac lipotoxicity can also participate in the cardiac functional consequences associated with obesity^4^. Lipids are important regulators of cardiac function through their role in membrane structure, cell transport, signaling and as substrate for β-oxidation for obtaining energy in the mitochondria^5,6^. Cardiac lipotoxicity not only involves an excessive accumulation of intra-myocellular triglycerides (TGs) in the heart but also changes in lipid classes, as well as in their fatty acid (FA) profile. Consequently, there is a regulation of the formation of active lipid mediators, which affect metabolism and cardiac function, in part by facilitating the development of cardiac fibrosis, an important contributor to heart muscle dysfunction^7,8^.

Acute myocardial infarction (MI), a major contributor of ischemic heart disease, undergoes extensive changes characterized by accumulation of extracellular matrix in both the infarcted and non-infarcted myocardium^9,10^. This cardiac remodeling alters tissue structure and increases tissue stiffness that facilitates ventricular dysfunction. Overweightness and obesity have a well-known role in the occurrence of MI^11^. However, the structural changes that occur in ischemic myocardium might be different in the context of obesity^12,13^.

MicroRNA (miRNA) are small non-coding RNAs which influence cell function by modulating gene expression mainly at the posttranscriptional level by either degrading the target mRNAs or inhibiting their translation. miRNAs are not only expressed at intracellular level but also are detected in biological fluids as circulating miRNAs, which have been suggested as potential diseases markers^14,15^. Several studies have supported a variety of roles of miRNAs in cardiac damage and repair since they can affect cardiac regeneration, energy homeostasis or cytoskeletal proteins. In this context, changes in miRNAs signature have been suggested as possible predictors of evolution of different cardiac pathologies^16,17^. Therefore, the aim of this study was to explore whether changes in the lipid cardiac profile could reflect differences in cardiac function and structure in obese and non-obese rats with MI. In addition, we explore if these changes can be reflected in a different miRNAs signature in obese and non-obese rats in the presence of myocardial ischemia.

## Methods

### Experimental design and animals

Male Wistar rats of 150 g (Harlan Ibérica, Barcelona, Spain) were fed either a high-fat diet (HFD, 35% fat; Harlan Teklad number, TD.03307, MN; n = 10) or a standard diet (3.5% fat; Harlan Teklad number, TD.2014, MN; n = 8) for 10 weeks. At the end of the six week, once a significant difference in body weight was observed between groups, MI was induced by ligation of the left anterior descendent (LAD) coronary artery. Briefly, the rats were anesthetized (2% isofluorane), intubated and ventilated (Inspiraasv, Harvard Instruments) and placed on an adjustable heating pad to maintain a core temperature of 36–37 °C). The heart was exposed through the fourth intercostal space separated with an adjustable microretractor (Medicon eG, Tuttlingen, Germany) and LAD ligated using 6/0 silk suture (Ethicon Endo-surgery, OH, USA), 1 mm distal to left atrial appendage. A third group of rats fed with a standard diet and subjected to SHAM operation (the same surgical procedure except that the suture passing the LAD was not fastened; n = 8) was included as a reference group (CT). After surgery, buprenorphine (0.05 mg/kg per 8 h, intramuscular) was given for 48 h. After recovery, the animals were kept in collective cages with free access to food and water. Systolic blood pressure (SBP) was estimated end-of-study through use of a tail-cuff plethysmograph (Narco Bio-Systems) in unrestrained rats. Animals were sacrificed by decapitation 4 weeks after the surgery. The Animal Care and Use Committee of Universidad Complutense de Madrid approved all experimental procedures according to the Spanish Policy for Animal Protection RD53/2013, which meets the European Union Directive 2010/63/UE.

### Biochemical parameters

Blood was collected in EDTA tubes and abdominal adipose tissue was dissected and weight. Adiposity index was calculated as sum of fat pads/(body weight-fat pad weight) × 100). Plasma leptin and insulin levels were measured using a specific quantitative sandwich enzyme immunoassay according to the manufacturer’s instructions (Biovendor, Germany Chemical Company and Mercodia AB; Uppsala;Sweden, respectively). Plasma concentrations of glucose, total cholesterol, and triglycerides were determined using spectrophotometric techniques in an autoanalyzer (Vitros 5600, Diagnostics Ortho Clinical, Johnson & Johnson, New Brunswick, NJ, USA). Insulin resistance was assessed by homeostasis model assessment of insulin resistance index (HOMA-IR), calculated from the formula^18^:

$$\begin{array}{rcl}\mathrm{HOMA} \mbox{-} \mathrm{IR} & = & {\rm{fasting\; glycemia}}\,(\mathrm{mmol}/{\rm{L}})\,\times \,{\rm{fasting\; insulinemia}}\,(\mathrm{mIU}/\mathrm{mL})/\mathrm{22.5.}\end{array}$$

### Evaluation of cardiac structure and function

#### Echocardiographic study

Cardiac function was evaluated by transthoracic echocardiography with an Acuson Sequoia 256 (Siemens Medical Solutions, Germany) connected to a 15-MHz linear transducer. 2D-guided M-mode recordings were made from short axis views to measure left ventricular (LV) chamber dimensions, interventricular septum and posterior wall thickness. LV diastolic (LVDA) and systolic (LVSA) areas were measured from the bidimentional parasternal long-axis view. The mean measurements from several consecutive beats were used for data analysis. LV chamber volumes were calculated using the cylindrical model. This model assumes the ventricle is approximated by a cylinder. Left ventricular ejection fraction (EF) was calculated according to the Teicholz Formula: (EDD^3^ × 7)/(2.4 + EDD) and LV systolic chamber function was determined from LV endocardial fractional shortening (FS) = (LVEDD-LVESD)/LVEDD × 100. The diastolic function was assessed by early and late transmitral peak diastolic flow velocity (E and A waves) and ratio between E-waves and A-wave (E/A) was calculated. The images were processed with the software MASS (Medis Medical Imaging Systems, Best, Norway).

#### NRM study

NMR study was performed with a Biospec BMT 47/40 spectrometer (Bruker, Ettlingen, Germany), located at the NMR Center of the Universidad Complutense of Madrid, equipped with a 12-cm gradient system and connected to a 1025 SAM monitoring and gating system (SA Instruments, Inc., New York, USA). At the beginning of the study, animals received i.p. Gd-based contrast agent (0.8 ml; MultiHance 529 mg/ml, Bracco Imaging, Milan, Italy).

Repetition time (TR) was variable depending on the animal’s heart and respiration rate. Other parameters were: echo time (TE) = 2.7 ms; Flip angle (θ) = 80°; Field of view (FOV) = 6 × 6 cm^2^; Slice thickness = 2 mm; Matrix size = 128 × 128; Number of averaged images = 2.

Once the short axis is set, a multislice T_1_ weighted bright-blood FLASH (Fast low angle shot) sequence was used to measure the infarct volume. The images were cardiac and respiratory triggered and the TR therefore varied depending on the animal’s heart rate. The averaged TR was 169 ms. Only one image, at the end of the diastolic phase, per cardiac cycle was acquired. Other parameters were: TE = 2.2 ms, θ = 90°, FOV = 5.12 × 5.12 cm^2^; Slice thickness = 2.0 mm; Matrix size = 256 × 128; and the number of averaged images was 4. The acquired data were zero-filled in the phase direction to achieve a reconstructed matrix size of 256 × 256. 7 or 8 slices were acquired to cover the whole heart.

In these images, the infarcted myocardial tissue appears brighter than the normal tissue. The infarct volume was calculated using ParaVision 3.1 (Bruker, Ettlingen, Germany). Regions of interest (ROI) was manually drawn in each slice that shows an infarcted area and the volumes of each ROI were added together to get the total infarct volume. Values were in mass units, multiplying them by the rat myocardial density (1.053 g/ml).

Images were processed with the software MASS (Medis Medical Imaging Systems, Best, Norway). The LV myocardium was delimited by endocardial and epicardial contours, which were traced manually.

### Morphological and histological evaluation

Cardiac tissue samples were dehydrated, embedded in paraffin and cut in 4 μm-thick sections. Sections were stained with picrosirius red in order to detect collagen fibers. The area of cardiac interstitial fibrosis was identified as the ratio of interstitial fibrosis or collagen deposition to the total tissue area after excluding the vessel area from the region of interest. For each sample, 10 to 15 fields were analyzed with a 40X objective under transmitted light microscopy (Leica DM 2000; Leica AG, Germany).

### Isolation of cardiac mitochondria

Cardiac mitochondria were isolated as reported^19^. Frozen hearts were placed and washed in cold homogenization medium containing 0.075 mol/L sucrose, 1 mmol/L EDTA, 10 mmol/L Tris–HCl, pH 7.4. Briefly, heart tissue was homogenized (1:10 *w*/*v*) at 800 rpm in a homogenizer (T 10 basic Ultra-turrax, Ika-Werke; Germany). The homogenates were centrifuged at 1,300 g for 5 min at 4 C to remove nuclei and debris. Supernatants were separated and centrifuged at 12,000 g for 10 min at 4 °C. The resulting pellets were suspended in homogenization medium and centrifuged twice at 14,400 g for 3 min at 4 °C to wash the mitochondrial fraction. Mitochondrial pellets were stored at −80 °C until use. Protein concentration was determined by the Bradford method.

### Western blot

Cardiac proteins were separated by SDS-PAGE on polyacrylamide gels and transferred to Hybond-c Extra nitrocellulose membranes (Hybond-P; Amersham Biosciences, Piscataway, NJ). Membranes were probed with primary antibody for carnitine palmitoyl transferase I (CPT1A, Abcam, Cambridge; dilution 1/1000), fatty acid traslocase (FAT, Abcam, Cambridge; dilution 1/1000), adipose triglyceride lipase (ATGL; Abcam, Cambridge; dilution 1/1000), diacylglycerol O-acyltransferase 1 (DGAT1; Abcam, Cambridge; dilution 1/1000) ands glyceraldehyde 3-phosphate dehydrogenase (GAPDH,Sigma; dilution: 1:10,000) as a loading control. Signals were detected using the ECL system (Amersham Pharmacia Biotech). Results are expressed as an n-fold increase over the values of the control group in densitometric arbitrary units.

### Lipidomic analysis

Myocardial total and mitochondrial lipids were extracted and analyzed by ultrahigh performance liquid chromatography coupled to time-of-flight mass spectroscopy (UPLC-QToF-MS) using an Acquity UPLC System and a SYNAPT HDMS G2 (Waters, Manchester, UK) with electrospray ionization. Extraction of lipids was carried out from cardiac homogenates in methanol:chloroform mixture (1:2, v/v) and split into two aliquots. One aliquot was evaporated to dryness and the pellet re-suspended in acetone:2-propanol:ethanol (3:4:3, v/v/v) and used for TGs measurement. The other aliquot was evaporated to dryness and the pellet re-suspended in methanol:water (9:1, v/v/v) and used for phospholipids (PLs) measurement. Extracts were kept at −80 °C until analysis. Mass spectrometric analysis of TGs was performed in positive mode (ESI+) using the parameters that follow: capillary, 0.8 kV; sampling cone, 15 V; source temperature, 90 °C; desolvation temperature, 280 °C; cone gas, 40 L/h; and desolvation gas, 700 L/h. Data were acquired with the software MassLynx at a rate of 5 scans/s within the range 0–18 min, and m/z 100–1200 Da for the low-energy function and m/z 100–900 Da for the high-energy function (MS^E^ method, trap collision energy 30 V). LC and MS methods were optimized using the commercial standards TG (18:2/18:2/18:2) and TG (16:0/16:0/16:0). These standards were also used to draw calibration curves for quantification. Mass spectrometric analysis of PLs was fitted as follows: capillary, 0.9 kV; sampling cone, 18 V; source temperature, 90 °C; desolvation temperature, 320 °C; cone gas, 45 L/h; and desolvation gas, 900 L/h. Data were acquired with the software MassLynx at a rate of 5 scans/s within the range 0–12 min and 100–1200 Da m/z for the low-energy function, and 50–900 Da m/z for the high-energy function (MS^E^ method, trap collision energy 30 V), with ionization in positive mode (ESI+) for detection of diacyl phosphatidylcholines (PCs), ceramides (CER) and sphingomyelins (SM), and with ionization in negative mode (ESI-) for detection of other phospholipids, which were diacyl phosphatidylethanolamine (PE), diacyl phosphatidylinositol (PI), diacyl phosphatidylglycerol (PG), and phosphatidic acids (PA). External commercial standards, namely PI (8:0/8:0), PG (14:0/14:0), PE (12:0/12:0), PC (10:0/10:0) and PA (14:0/14:0) were purchased from Cayman Chemical (Michigan, USA) and used for method optimization and quantification.

Up to three different chromatograms were manually checked for mass spectral peak identification where possible. Within each chromatographic point, m/z values with an intensity >=700 were also checked for it in order to afford a defined chromatographic peak (Extracted Ion Chromatogram, EIC); if positive, the elemental composition tool was then used to determine all the possible chemical compositions (C_n_H_m_O_p_N_s_P_r_S_t_) that were compatible with the isotopic distribution (M, M + 1, M + 2 and M + 3 peaks) of a given m/z value. Using LipidMaps, Metlin, CheBI, LipidBank and KEGG databases, a certain elemental composition was examined for possible known compounds. Where possible, acyl chains were identified by data from the high-energy function (fragmentation). As well, specific fragments in the high energy function (MS^E^) were considered for identification, in particular m/z 184.07 for PCs and SMs in positive ionization mode.

### RNA isolation

A 250 µl EDTA plasma sample from each animal was used for RNA isolation using the Serum and Plasma miRNeasy kit (Quiagen, Hilden, Germany) according to the manufacturer’s instructions. RNA quality control was conducted using 260/280 ratios (only those with a ratio between 1.8–2.2 were included).

### PCR-array study

Isolated miRNA samples (10 ng per animal) from the 3 groups were divided into three pools (n = 6 animals each) in order to quantify miRNAs expression using microRNA Human PCR panels (Exiqon). Briefly, 30 ng of total RNA from each pool were employed for cDNA synthesis with the miRCURY LNA™ Universal RT microRNA PCR System (Exiqon) following the manual’s instructions. Reverse transcription reaction efficiency and polymerase chain reaction (PCR) inhibitors presence were checked with UniSp6 and cel-miR-39–3p, respectively. PCR determinations were then performed using Serum/Plasma Focus microRNA PCR Panel (V4.M) (Exiqon). This panel allows for the analysis of 179 miRNAs. The amplification was performed in an ABI 7900HT qPCR instrument (Life Technologies Ltd.) in 384-well plates. The amplification curves were analyzed using the SDS v.2.4 software (Life Technologies Ltd.) with manual baseline/threshold settings to determine the threshold cycles (Ct). Water control assays in which DNA was omitted were included to assess the presence of contamination. In order to identify a plasma expression miRNA profiling, an initial selection from the 179 human miRNAs analysed was performed by eliminating 28 miRNAs since they were not expressed in any group. Next, all values showed as “undetermined” were substituted by 35 as Ct, and data were normalized using miR-23a-3p as reference control, which was previously selected from a panel screening of stably expressed miRNAs (Fig. S1). Then, fold changes were calculated using 2−ΔΔCq method^20^.

### Quantitative real-time PCR analysis

Eleven miRNAs (7-1-3p, 15a-5p, 15b-5p, 19a-3p, 29b-3p, 34a-5p, 144-5p, 194-5p, 301a-3p, 1260a and let7f-5p) which show differential expression among the 3 groups were selected for quantitative real-time PCR (qRT-PCR) validation (Table S1). For each individual isolated miRNA sample, 10 ng of total RNA were employed for cDNA synthesis as described above and qRT-PCR was then performed using SYBR® Green master mix and specific LNA™ PCR primer sets (Exiqon) for each miRNA of interest following manufacturer’s instructions. As previously mentioned, miRNA expression data were calculated using ΔCt method with miR-23a-3p as internal control.

### Statistical analysis

Continuous variables are expressed as mean ± SEM. Normality of distributions was verified by means of the Kolmogorov–Smirnov test. One-way ANOVA was used and followed by Newman-Keuls test. Pearson correlation analysis was used to examine association among different variables according to whether they are normally distributed. Multivariable analysis, considering fibrosis as the dependent variable, was performed with a linear regression model by means of a backward stepwise method. In consecutive steps, variables that were statistically significant in the univariable analysis were included in the linear regression model. A value of P < 0.05 was used as the cutoff value for defining statistical significance. Data analysis was performed using the statistical program SPSS version 22.0 (SPSS Inc, Chicago, IL, USA).

## Results

Animals fed an HFD had higher body weight, adiposity index and plasma leptin levels than those fed a standard diet independently of the presence of MI (Table 1). In addition, plasma glucose levels and HOMA index were also increased in obese animals as compared to those fed a standard diet independently of the presence of MI, suggesting systemic insulin resistance (Table 1). No changes were observed in total cholesterol plasma levels among any group although an increase in plasma TG was found in HFD-AMI rats as compared with the other 2 groups (Table 1). No significant differences were observed in systolic blood pressure among any group (Table 1).

**Table 1:** Impact of myocardial infarction on general characteristics, relative heart weight LV mass and infarct size in rats submitted to myocardial infarction fed a standard diet (AMI) or a high fat diet (HFD-AMI) and rats fed a control diet and with SHAM operation (CT).

Parameter	CT	AMI	HFD-AMI
Body weight (g)	396.2 ± 10.8	394.8 ± 9.7	470.2 ± 13.8***^†††^
Adiposity (%)	6.5 ± 0.5	4.9 ± 0.5	11.1 ± 0.9***^†††^
Plasma Leptin levels (ng/ml)	2.2 ± 0.2	2.5 ± 0.4	20.3 ± 4.3***^†††^
HOMA	3.2 ± 0.5	2.5 ± 0.4	8.6 ± 1.2***^†††^
Plasma Glucose levels (mg/dl)	111.8 ± 1.1	114.7 ± 4.4	130.0 ± 4.9*^†^
Plasma TC levels (mg/dl)	71.8 ± 3.9	75.6 ± 4.5	83.3 ± 5.9
Plasma TG levels (mg/dl)	92.7 ± 8.7	84.6 ± 10.1	135.9 ± 10.5**^††^
SBP (mmHg)	127.72 ± 2.7	132.6 ± 1.3	131.0 ± 0.01.7
Heart relative weight (g/cm tibia)	0.229 ± 0.01	0.253 ± 0.008*	0.271 ± 0.003***
LV mass (g)	0.287 ± 0.061	0.420 ± 0.015***	0.477 ± 0.02***^†^
Infarct size/LV mass (%)	—	18.1 ± 2.9	20.7 ± 1.6

HOMA: Homeostatic model assessment; TC: total cholesterol; TG: triglycerides. SBP: Systolic blood pressure; LV: Left ventricle. Data are expressed as mean ± SEM. of 8–10 animals. *P < 0.05; **P < 0.01; ***P < 0.001 vs control group. ^†^*P* < 0.05; ^††^*P* < 0.01; ^†††^*P* < 0.001 vs AMI group.

**Figure 1:**
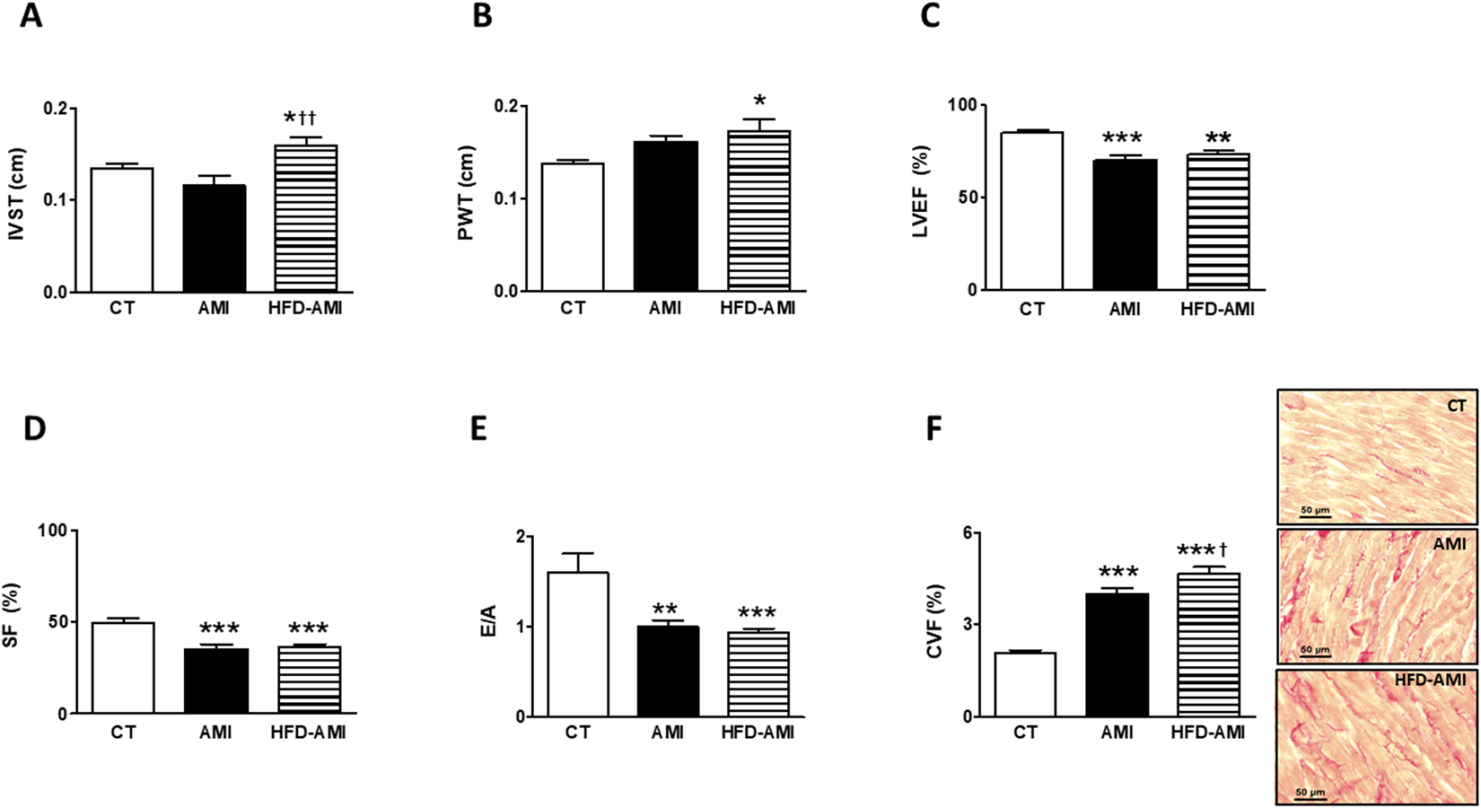
Impact of MI on cardiac echocardiographic parameters and interstitial fibrosis in non-obese and obese rats. (**A**) Septum interventricular thickening (IVST); (**B**) Posterior wall thickening (PWT); (**C**) Left ventricular ejection fraction (LVEF); (**D**) shortening fraction (SF); (**E**) E-wave and A-wave ratio (E/A); (**F**) Quantification of collagen volume fraction (CVF) and representative microphotographs of myocardial sections staining with picrosirius red examined by light microscopy (magnification 40x) in control rats (CT), or rats submitted to MI fed a standard (AMI) or a high fat diet (HFD-AMI). Bar graphs represent the mean ± SEM. of 8–10 animals. *P < 0.05; **P < 0.01; ***P < 0.001 vs control group. ^†^*P* < 0.05; ^††^*P* < 0.01 vs AMI group.

Infarct size was similar between obese and non-obese animals (Table 1). Compared with CT group, animals with MI showed a higher relative heart weight, suggesting cardiac hypertrophy, which was more prominent in obese than in non-obese animals. Left ventricle mass was also higher in both groups with MI (Table 1). Obese animals showed thickening of both interventricular septum (IVS) and posterior wall (PW) as compared with control animals (Fig. 1A,B). No differences were observed in left ventricular end-diastolic diameter (LVEDD) among the three groups (Fig. S2A). However, MI induced an increase in left ventricular end-systolic diameter (LVESD) independently of the type of diet (Fig. S2B). Left ventricular ejection fraction (LVEF), shortening fraction (SF) and the E/A ratio were reduced in animals with MI (Fig. 1C–E). Animals with MI also showed an increase in interstitial fibrosis as compared with CT group, which was larger in obese than non-obese rats (Fig. 1F). As shown in Table S2, fibrosis was associated with E/A, LVEF and SF.

**Figure 2:**
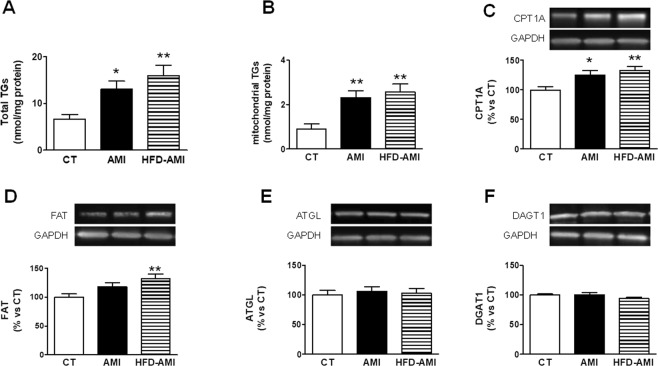
Impact of MI on total and mitochondrial triglycerides and protein levels in heart from non-obese and obese rats. Cardiac levels of (**A**) total triglycerides (TGs), (**B**) mitochondrial triglycerides. Protein expression of (**C**) carnitine palmitoyl transferase 1 (CPT1A), (**D**) Fatty acid translocase (FAT), (**E**) adipose triglyceride lipase (ATGL) and (**F**) diacylglycerol transferase 1 (DAGT1) in control rats (CT), or rats submitted to MI fed a standard (AMI) or a high fat diet (HFD-AMI). Bar graphs represent the mean ± SEM. of 8–10 animals normalized to glyceraldehyde-3-phosphate dehydrogenase (GAPDH). *P < 0.05; **P < 0.01; ***P < 0.001 vs control group. ^†^ < 0.05; ^††^*P* < 0.01; ^†††^*P* < 0.001 vs AMI group.

Cardiac lipidomic analysis showed an increase in total TG content in the heart, which was slight higher in obese animals (Fig. 2A). This increase was also observed at mitochondrial level where TGs increased 2.5 and 2.8 fold in the non-obese and obese rats with MI, respectively, as compared with CT group (Fig. 2B). As shown in Table S2, mitochondrial TGs were associated with systolic and diastolic function.

We explored whether high cardiac TG levels could be due to an increase in inflow into cell and mitochondria. Figure 2C shows an increase in CPT1A levels in both obese and non-obese rats with MI, which was larger in the obese animals. However, FAT levels were only increased in obese animals with MI (Fig. 2D). No differences in both ATGL and DGAT1 levels–involved in the last step of TG synthesis or intracellular degradation of TG, respectively–were observed among any group (Fig. 2E,F).

**Figure 3:**
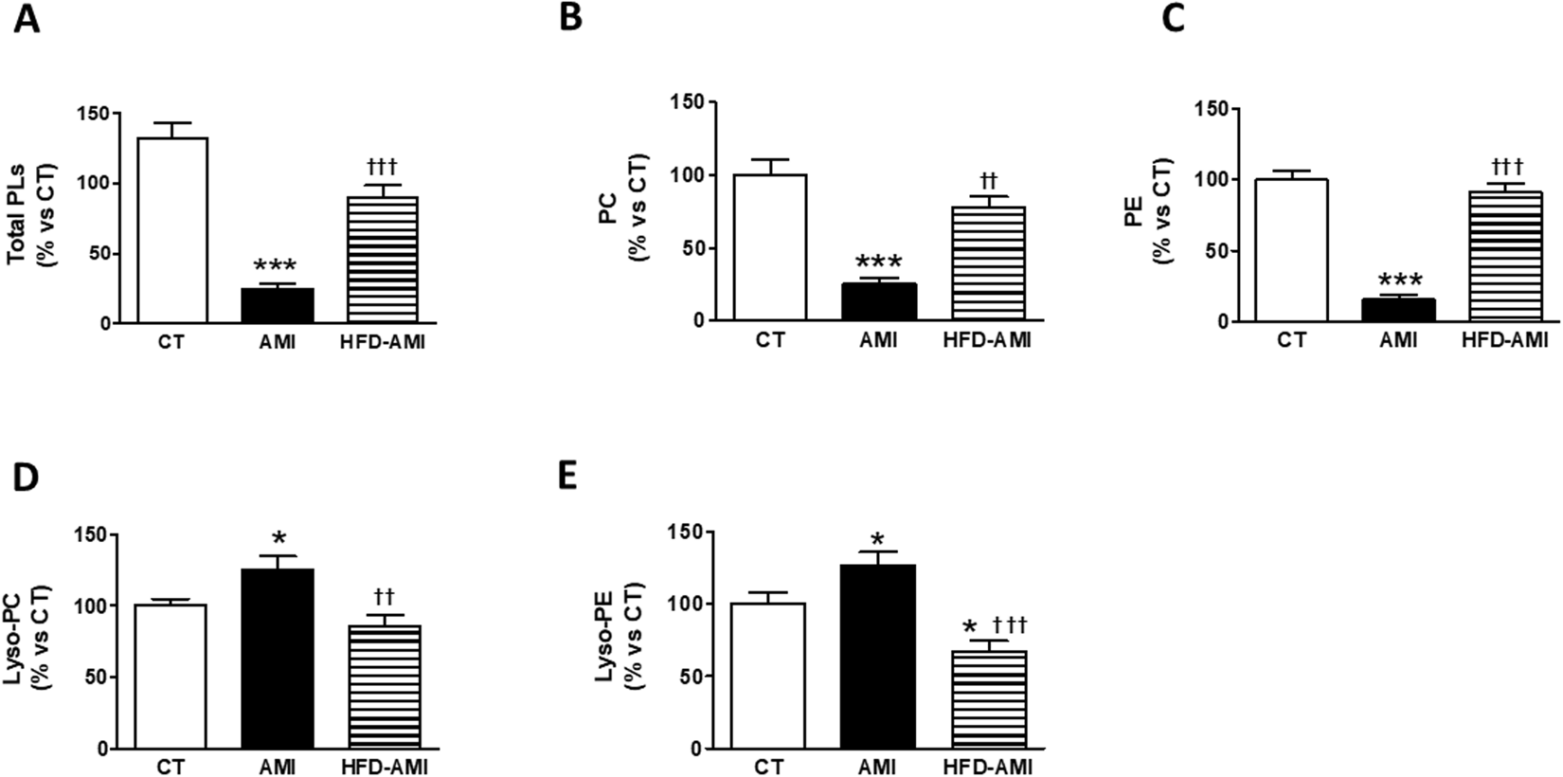
Impact of MI on mitochondrial lipid species in heart from non-obese and obese rats. Cardiac levels of (**A**) total phospholipids (PL); (**B**) diacyl phosphatidylcholine (PC); (**C**) diacyl phosphatidylethanolamine (PE); (**D**) Lysophosphatidylcholine (Lyso-PC) and (**E**) Lysophosphatidylethanolamine (Lyso-PE) in control rats (CT), or rats submitted to MI fed a standard (AMI) or a high fat diet (HFD-AMI). Bar graphs represent the mean ± SEM. of 8–10 animals normalized to CT group. *P < 0.05; ***P < 0.001 vs control group; ^††^*P* < 0.01, ^†††^*P* < 0.001 vs AMI group.

Considering this important increase in the cardiac mitochondrial TGs, we explore whether myocardial ischemia can affect the lipid molecular species profile of mitochondria. Obese animals with MI did not show any significant changes in either mitochondrial levels of total PL, total diacyl phosphatidylethanolamine (PC) or total diacyl phosphatidylcholine (PE) in the heart as compared with CT (Fig. 3A–C, respectively). However, a reduction in total mitochondrial PLs levels was observed in non-obese rats with MI as compared with CT animals (Fig. 3A). This reduction was mainly due to the low levels in cardiac mitochondrial PC (Fig. 3B) and PE (Fig. 3C). Detailed evaluation of each lipid class revealed that the cardiac mitochondrial PE-species significantly down-regulated in non-obese rats with MI and were those mostly containing the polyunsaturated long-chain FAs 20:4 and 22:6 (Fig. S3A,B). Mitochondrial PC-species were also differentially regulated in ischemic-non obese hearts: PC-species were also mainly depleted from the FAs 20:4 (Fig. S3C). An increase in either cardiac mitochondrial Lysophosphatidylcholine (Lyso-PC) or Lysophosphatidylethanolamine (Lyso-PE) levels were observed in non-obese infarcted rats as compared with CT and obese rats with MI (Fig. 3D,E) mainly due to those being enriched with saturated acids (18:0; Fig. S4A,B), as well as with 20:4 (Fig. S4C,D). Interestingly, Lyso-PE levels were reduced in obese rats as compared with control ones (Fig. 3E).

**Figure 4:**
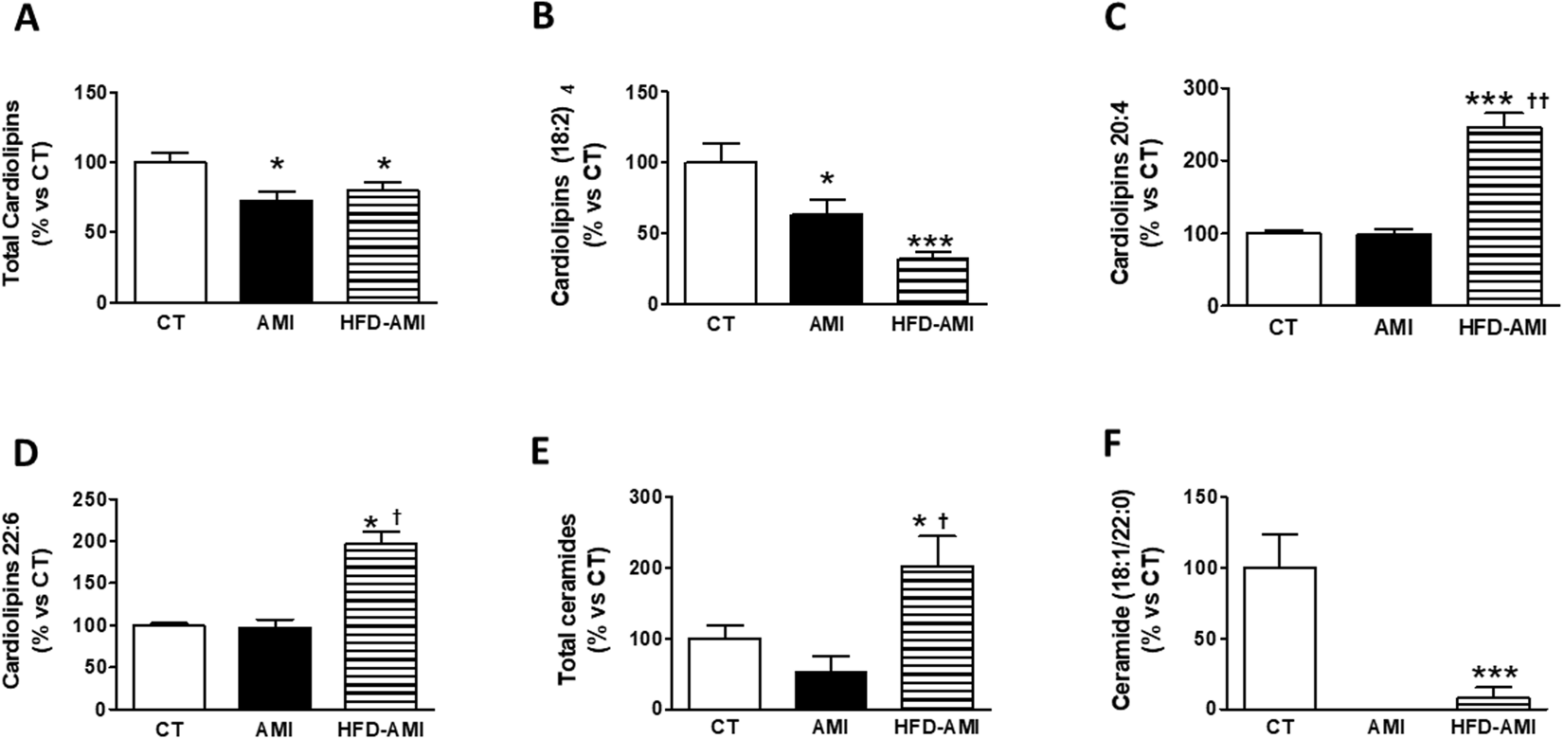
Impact of MI on mitochondrial cardiolipins and ceramide species in heart from non-obese and obese rats. Cardiac levels of (**A**) total cardiolipins; (**B**) cardiolipins enriched with linoleic acid; (**C**) cardiolipins enriched with arachidonic acid (20:4), (**D**) cardiolipins enriched with docosahexaenoic acid (22:6), (**E**) Total ceramides and (**F**) ceramides enriched with docosanoic acid (22:0). Bar graphs represent the mean ± SEM. of 8–10 animals normalized to CT group. *P < 0.05; ***P < 0.001 vs control group. ^†^*P* < 0.05; ^††^*P* < 0.01vs AMI group.

Mitochondrial cardiolipins (CL) levels in heart were also reduced in the non-obese and obese animals (Fig. 4A) with MI due to the reduction in those enriched with 18:2, especially the tetra one (18:2)_4,_ which was the most abundant species detected (Fig. 4B). Interestingly, cardiac mitochondrial levels of both CL 20:4 and 22:6 were increased in rats with MI fed an HFD (Fig. 4C,D). No changes in cardiac mitochondrial carnitine or sphingomyelins levels among any group were found (Fig. S5A,B). However, the overall cardiac ceramides(CER) levels in mitochondria were elevated in obese infarcted animals as compared with the other two groups (Fig. 4E) and were correlated with HOMA (r = 0. 672; p = 0.003) and adiposity (r = 0.663; p = 0.004). All the long-chain cardiac CER (especially 16:0; Fig. S5C) were found to be upregulated in the mitochondria from HFD fed rat with MI, with the exception of the very-long-chain C22 species, which dramatically decreased (Fig. 4F).

The mitochondrial lipid species associated with fibrosis were defined by a linear regression analysis as shown in Table 2. The independent association of blood pressure, obesity and metabolic alterations was tested. Independent predictors of fibrosis were cardiac mitochondrial TGs (mean difference, 0.89; 95% CI, 0.48–1.31; *P* = 0 0.001).

**Table 2:** Association between levels of myocardial fibrosis and those of cardiac lipid species, plasma miRNAs, adiposity and leptin in rats submitted to myocardial infarction fed a standard diet (AMI) or a high fat diet (HFD-AMI) and rats fed a control diet and with SHAM operation (CT).

Variable	Beta coefficient	95% confidence interval	p
Total TG	0.156	0.06–0.25	0.003
Mitochondrial TGs	0.99	0.55–1.44	<0.001
Adiposity	0.18	0.03–0.32	0.017
Leptin	0.00	0.00–0.00	0.011
Total CL	−0.19	−0.33–0.05	0.010
CL (22:6)	1.71	0.18–3.23	0.030
CL (20:4)	0.49	0.11–0.87	0.015
CL (18:2)_4_	−0.31	−0.48–0.14	0.001
CER (22:0)	−15.79	−25.78–5.79	0.004
miRNA 194-5p	−2.30	−4.38–0.22	0.032
miRNA 301a-3p	−1.89	−3.42–0.36	0.032
miRNA 144-5p	−1.62	−2.82–0.41	0.012
miRNA 15b-5p	−2.05	−3.19–0.92	0.001

CER: ceramide; TGs. Triglycerides; CL: cardiolipins.

By profiling the pooled samples of CT and obese and non-obese rats with MI, we found that MI shows a plasma miRNA signature that was significantly different from that of CT. 151miRNAs were detected by PCR array where 16 miRNAs were increased and 7 miRNAs were decreased by more than two-fold in non-obese rats with MI compared to CT rats. This signature was further modified in the presence of obesity since, as compared with non-obese rats with MI, 8 plasma miRNAs were increased and37 miRNAs were decreased by more than two-fold. Therefore, we selected 11 most significantly differentially expressed miRNAs among the three groups (Table S1).

**Figure 5:**
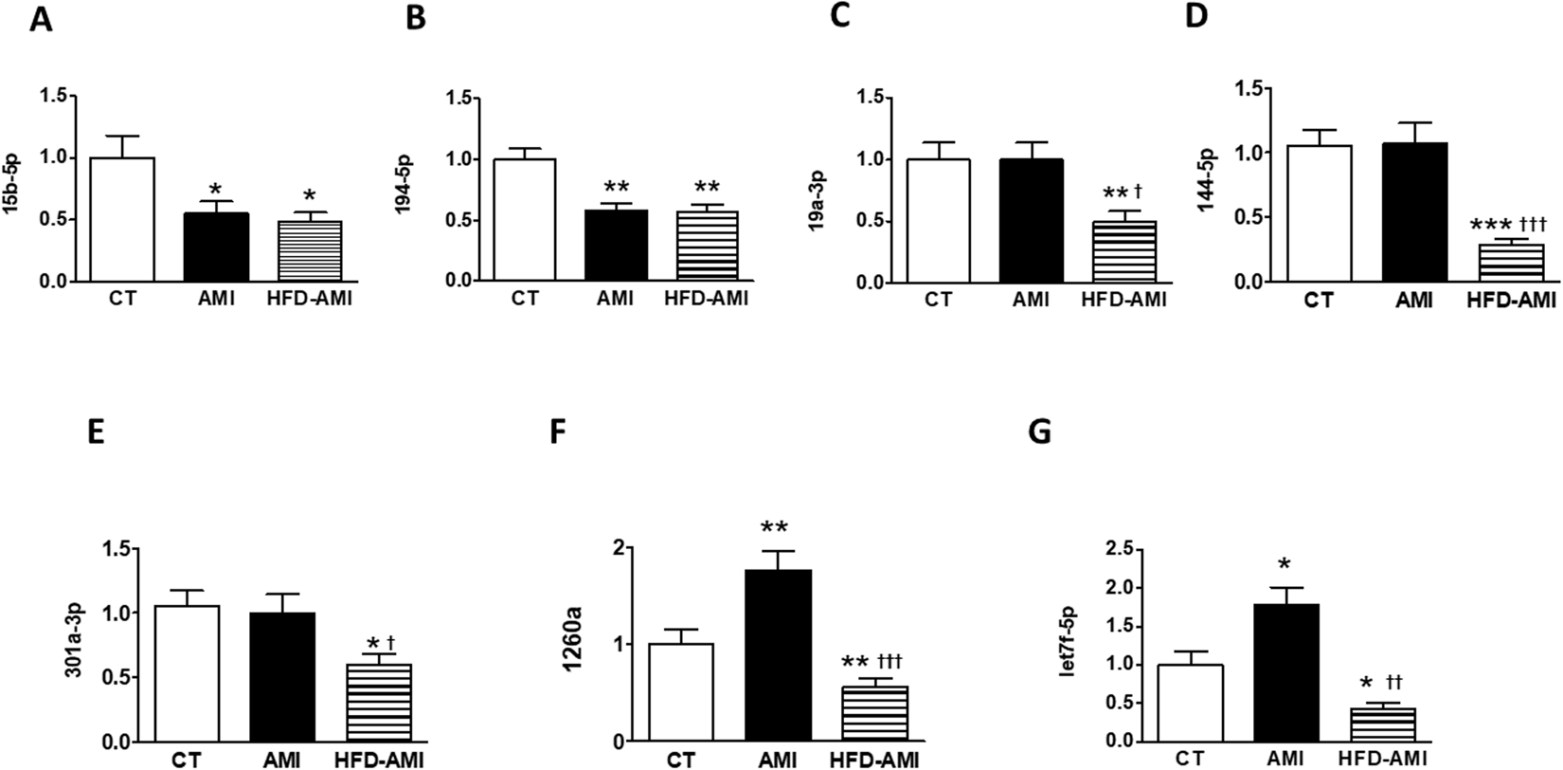
Impact of MI on plasma-derived microRNAs from non-obese and obese rats. Plasma levels of (**A**) microRNA (mRNA) 15b-5p; (**B**) mRNA 194–5p; (**C**) mRNA 19a-3p; (**D**) mRNA 144-5p; (**E**) mRNA 301a-3p; (**F**) miRNA1260a and (**G**) mRNA let7f-5p in control rats (CT), or rats submitted to MI fed a standard (AMI) or a high fat diet (HFD-AMI). Bar graphs represent the mean ± SEM. of 8–10 animals normalized to CT group. *P < 0.05; **P < 0.01; ***P < 0.001 vs control group. *P* < 0.05; ^††^*P* < 0.01; ^†††^*P* < 0.001 vs AMI group.

MI down-regulated plasma levels of both miRNA 15b-5p and 194-5p independently of the diet as compared with CT rats (Fig. 5A,B). Their levels were correlated with systolic function (Table S2). In addition, plasma levels of miRNA 15b-5p or 194-5p were correlated with those of cardiac levels in mitochondria of both CL (18:2)_4_ and TG, respectively (Table S3). Obese rats with MI show a decrease in plasma levels of 19a-3p, 144-5p and 301a-3p as compared with animals of the other two groups (CT and AMI; Fig. 5C–E). All of these were associated with adiposity index (Table S4). In addition, plasma levels of miRNA 144-5p were associated with those of HOMA (Table S4). Their levels were associated with different CL species (Table S3). A different pattern in plasma levels of miRNA 1260a and let7f-5p was observed in obese and non-obese rats with MI since, as compared with CT group, they increased in non-obese infarcted animals but they were down-regulated in obese rats (Fig. 5F,G). Plasma levels of miRNAs 1260a and let7f-5p were correlated with cardiac levels in mitochondria of both Lyso-PC and Lyso-PE (Table S5). No significant changes were observed in plasma miRNAs levels of either 15a-5p, 7-1-3p, 29b-3p or 34a-5p among any group (Fig. S6A–D).

The plasma levels of miRNAs associated with fibrosis were defined by a linear regression analysis as shown in Table 2. The independent association of blood pressure, obesity and metabolic alterations was tested. One independent predictor of fibrosis was plasma levels of miRNA 15b-5p (mean difference, −1.13; 95% CI, −2.09– −0.17; *P* = 0 0.025).

## Discussion

Cardiac lipotoxicity is a well-established feature in the context of obesity and is associated with cardiac damage. However, whether MI can affect/modify lipid profile is unknown. We herein report that MI is accompanied by cardiac lipotoxicity even in the absence of obesity and seems to have functional consequences. However, obese and non-obese animals show a differential lipid species pattern at mitochondrial level. In addition, MI induces a specific plasma miRNA signature that was further modified in the presence of obesity and is associated with a specific myocardial lipid profile.

The data show an increase in cardiac TG in obese rats, which was also observed at mitochondrial level and supports cardiac lipotoxicity, a well-established feature in the context of obesity or diabetes^21–23^. Surprisingly, cardiac lipotoxicity was also observed in non-obese animals with MI even in the absence of changes in the plasma cholesterol and TG levels or glucose homeostasis, suggesting that MI is accompanied by lipid accumulation in the heart. More important, this effect has functional consequences since cardiac TG levels were independently associated with fibrosis, which affects cardiac contractility and relaxation. This can explain the observation that TG were associated with both systolic and diastolic function, supporting the relevance of cardiac lipotoxicity in cardiac dysfunction. These data agree with the observation that intracellular lipid accumulation in the myocardium of type 2 diabetic patients is associated with alterations of contractile mechanisms, a reduced capacity of myocytes towards ischemia and higher susceptibility to death as a result^23^. An increased cellular and mitochondrial FA uptake could be involved in the high TG levels observed since a rise in both FAT and CPT1A–proteins involved in the uptake and intracellular trafficking of FA acids–was observed in animals with MI, confirming previous observations^7^. By contrast, no changes were observed among any group in either DGAT1 or ATGL enzymes involved in the last step of TG synthesis and intracellular degradation of TG, respectively, suggesting that changes in either TG metabolism seems not to be major contributors to the high TG levels.

CER, toxic lipid intermediates that can cause cellular dysfunction by affecting mitochondrial function^24,25^, increased their levels only in mitochondria of heart from obese rats with MI. Although mitochondrial CER levels were elevated in obese rats overall, the levels of C22:0-ceramide - with protective effect against hypoxia^26^ -were selectively down-regulated in both groups with MI and correlated with fibrosis.Therefore, the imbalance between mitochondrial CER of different chain length may generate a more harmful cardiac environment, especially in the context of obesity. In addition, the high CER levels observed in obese rats with MI could be underlying the more marked cardiac remodeling observed in obese animals with MI, since they show a larger LV wall thickness compared with normoweight rats independently of the presence of ischemia, confirming previous observations^12,27,28^. This participation could be through their role in insulin resistance since alterations in the glucose homeostasis is a comorbidity frequently present in obese patients which can affect LV geometry^29^.

Our data also showed that MI was also accompanied by a reduction in total cardiac mitochondrial CL levels independently of the diet, supporting that hypoxia inhibits CL synthesis^30^. CL reduction is also observed in other pathological situations including obesity and heart failure^31–33^ and can occur even before TG accumulation^33^. CL content is critical for preservation of mitochondrial oxidative phosphorylation and inner membrane integrity, and energy production as a result^34^ Not only CL reduction levels but also their acyl chain remodeling can affect mitochondrial activity and cell function in consequence. Although overall cardiac mitochondrial CL were reduced, the levels of CL (20:4) and CL (22:6) were increased in obese rats with MI. Their levels were associated with larger IVS and PW thickness (data not shown), suggesting a role of these CL in the larger LV mass observed after MI in the context of obesity.

PL metabolism is also markedly affected by MI. A reduction of both total PC and PE, paralleled by a rise in Lyso-PE and Lyso-PC species, was observed in heart from normweight rats with MI but not in obese ones with MI ones at mitochondrial level, confirming previous observations in which ischemia/reperfusion was associated with a similar decrease in PLs levels in perfused heart from rabbits and rats^35,36^. The protective effect of obesity against these changes is unclear but can have functional consequences since mitochondrial PL are necessary for the proper localization and function of key mitochondrial enzyme^37^ and to myocardial contractility and viability as a result. The high PC/PE breakdown and the consequent increase in Lyso-PC/Lyso PE levels–mainly to those enriched with saturated acids (16:00 and 18:0), as well as with 20:4–may be triggered by an accelerated phospholipid catabolism caused by phospholipases activation^5,35^. Overall, these findings may support a differential role in obese and non-obese myocardium to the mitochondrial iPLA_2_γ. This PLA_2,_ which also possesses PLA_1_ activity, generates 20:4-LPC^38^ and contributes to the opening mitochondrial permeability transition pore, which induces cadiomyocyte dysfunction and death, thus leading to heart failure^39^. Notably, 20:4-Lyso-PC is the most abundant lysolipid molecular species in failing human hearts and serves as a central branch point metabolite in several inflammatory signaling cascades^40^.

Our data also show that MI, independently of presence or not of obesity, down-regulated miRNAs 15b-5p and 194-5p levels, suggesting a protective effect in the animals with MI. This affirmation is based on the fact that miRNA15b-5p negatively modulates angiogenesis, reducing collateral artery formation^41^. In fact, its levels were independently associated with myocardial fibrosis, supporting the relevance of the miRNA 15b-5p as a key regulator of the events triggered in response to hypoxia. The specific role of miRNA 194-5p is not well established, although high levels in patients that developed heart failure after MI have been reported and it has been suggested as a predictive indicator of heart failure after MI^42^.

Two different patterns in miRNA signature were observed in obese rats as compared with the non-obese ones independently or not of the presence of MI. The first one included miRNAs 19a-3p, 144-5p and 301a-3p, which were down-regulated in the obese rats as compared to the other two groups, suggesting a specific modulation of obesity. In fact, an association between adiposity and each of these miRNAs was observed. Moreover, we have found an association between HOMA levels and miRNA 144-5p, confirming previous observations, which implies it to be a potential regulator of insulin sensitivity in obese patients or patients with type 2 diabetes^43^. In addition, and confirming previous observations^44,45^, our data support a potential role of miRNA 19a-3p as a negative regulator of cardiac hypertrophy. A similar role for the miRNA 144-5p can be suggested that, in addition to its ability to modulate oxidative stress^46^, could explain the observation that lower levels of miRNA 144-5p have been associated with chronic heart failure or future MI^47^. The specific role of miRNA 301a-3p in our model is unclear, although a modulatory role of inflammatory/immune response in different scenarios^48,49^ has been suggested.

A second very different pattern was observed in the case of miRNAs lef7f5p and 1260a in animals with MI: an upregulation in non-obese rats with a down-regulation in obese rats as compared with normal rats. This supports an important impact of obesity on the expression of miRNAs in the context of MI. Different studies have reported a protective role of miRNA let-7f in response to ischemia/reperfusion by reducing brain infarct volume^50^ or reducing apoptosis in cardiomyocytes or mice by regulating insulin-like growth factor-1^50^. Therefore, if we consider our data in this context, it seems that this potential beneficial effect, which was observed in non-obese rats, disappears in obese ones. The potential role of miRNA 1260a is unclear since not so much information has been reported.

The significance/relevance of the fact that these miRNAs are associated with different lipid species, including cardiac mitochondrial CL and TGs, Lyso-PC or Lyso-PE, is unclear at this moment but it is possible to speculate that changes in cardiac lipid profile can be reflected in the miRNA signature. Correlations between lipid metabolism and several miRNAs has already been described, including miRNA122, miR-33/33*, miR27a/b, miR378/378*, miR-34a and miR-21^51^. In fact, circulating miR-122 is considered a marker of hepatic lipid metabolism. These data support that cardiac lipotoxicity could modulate miRNA profile and could be one potential mechanism through which cardiac lipotoxicity can affect cardiac function. Moreover, these different profiles of miRNAs and mitochondrial lipid observed between obese and non-obese rats with MI might serve as a tentative mechanistic explanation for the differences observed between both groups. In addition, they might determine different evolution and/or consequences of the MI in the context of obesity, although further investigation is required.

In summary, lipotoxicity is a well-known feature of the obese heart, which could be involved in the cardiac damage observed in obese patients. However, it seems that MI can also facilitate lipid accumulation in the heart through mechanisms unknown at the moment but can have functional consequences, since cardiac mitochondrial TG were independently associated with myocardial fibrosis. The lipid profile at mitochondrial level is not the same in obese and non-obese animals and could potentially determine a different evolution to MI in these two animal models over time. Moreover, obesity is associated with different circulating miRNAs profiles in the context of MI and they are associated with some lipid species. This could support that lipotoxicity could affect heart function by modulating miRNAs.

## Supplementary information

Supplementary data
