# Supplementary material for: The Impact of Cardiac Lipotoxicity on Cardiac Function and Mirnas Signature in Obese and Non-Obese Rats with Myocardial Infarction
Source: Sci Rep. 2019 Jan 24;9:444. doi: 10.1038/s41598-018-36914-y (PMC6345821; doi:10.1038/s41598-018-36914-y)
Supplement: Supplementary file 1 — Supplementary data [file 41598_2018_36914_MOESM1_ESM.docx]

**ONLINE SUPPLEMENT**

**THE IMPACT OF CARDIAC LIPOTOXICITY ON CARDIAC FUNCTION AND MIRNAS SIGNATURE IN OBESE AND NON-OBESE RATS WITH MYOCARDIAL INFARCTION**

Gema Marín-Royo^1^, Adriana Ortega-Hernández^2,3^, Ernesto Martínez-Martínez^1^, Raquel Jurado-López^1^, María Luaces^4^, Fabián Islas^4^, Dulcenombre Gómez-Garre^2,3^, Beatriz Delgado-Valero^1^, Esther Lagunas^1^, Bunty Ramchandani^5^, Mónica García-Bouza^6^, María Luisa Nieto^3,7^*, Victoria Cachofeiro^1,3^*.

^1^Departamento de Fisiología, Facultad de Medicina, Universidad Complutense de Madrid and Instituto de Investigación Sanitaria Gregorio Marañón (IiSGM), Madrid, Spain.^2^Laboratorio en Biología Vascular, Hospital Clínico San Carlos-IdISSC, Madrid, Spain.^3^Ciber de Enfermedades Cardiovasculares (CIBERCV). Instituto de Salud Carlos III. Madrid. Spain. ^4^Servicio de Cardiología, Instituto Cardiovascular, Hospital Clínico San Carlos, Madrid, Spain.^5^Departamento de Cirugía Cardiaca, Hospital Clínico San Carlos, Madrid, Spain. ^6^Departamento de Cirugía Cardiaca, Hospital Universitario de Canarias, Tenerife, Spain.^7^Instituto de Biología y Genética Molecular, CSIC-Universidad de Valladolid, Valladolid, Spain.

* These authors contributed equally to this work.

**Corresponding author:** V. Cachofeiro, Departamento de Fisiología, Facultad de Medicina, Universidad Complutense, Madrid 28040. Spain.

Phone: 34 913941489; FAX: 34 913941628. E-mail: vcara@ucm.es

This file includes:

7 supplemental figures

5 supplemental tables

**Figure S1.** Expression levels of several miRNAS in pooled samples. We tested eight candidates for reference gene, which were selected among genes that may be stably expressed in plasma/serum samples based on the literature and pre-existing data (Guidelines for the miRCURY LNA™ Universal RT microRNA PCR system, V2.0, Exiqon). PCR quantification cycle (Ct) values are presented. miRNA-23a-3p showed to itself to be a good candidate to be a reference gene for normalization.

**Figure S2. Impact of myocardial infarction on cardiac echocardiographic parameters in non-obese and obese rats.** Rats submitted to myocardial infarction were fed a standard diet (AMI) or a high fat diet (HFD-AMI). Rats fed a control diet and with SHAM operation were used as reference control group (CT). (A): left ventricle end diastolic diameter (LVEDD) and (B): left ventricle end systolic diameter (LVESD). Bar graphs represent the mean±SEM of 8-10 animals. ***P<0.001 vs control group.

**Figure S3. Impact of MI on mitochondrial lipid species in heart from non-obese and obese rats. Cardiac l**evels of (**A**) diacyl phosphatidylethanolamine (PE) enriched with arachidonic acid (20:4); (**B**) PE enriched with docosahexaenoic acid (22:6) and (**C**): diacyl phosphatidylcholine (PC) enriched with arachidonic acid (20:4) in control rats (CT), or rats submitted to MI fed a standard (AMI) or a high fat diet (HFD-AMI). Bar graphs represent the mean±SEM. of 8-10 animals normalized to CT group. **P<0.01; ***P<0.001 vs control group. ††P<0.01vs AMI group.

**Figure S4. Impact of MI on mitochondrial lipid species in heart from non-obese and obese rats. Cardiac l**evels of (**A**) Lysophosphatidylcholine (Lyso-PC) enriched with stearic acid (18:0); (**B**) Lysophosphatidylethanolamine (Lyso-PE) enriched with stearic acid (18:0); (**C**): Lyso-PC enriched with arachidonic acid (20:4) and (**D**) Lyso-PE enriched with arachidonic acid (20:4) in control rats (CT), or rats submitted to MI fed a standard (AMI) or a high fat diet (HFD-AMI). Bar graphs represent the mean±SEM. of 8-10 animals normalized to CT group. *P<0.05 vs control group. † P<0.05; ††P<0.01vs AMI group.

**Figure S5. Impact of MI on mitochondrial lipid species in heart from non-obese and obese rats. Cardiac l**evels of (**A**) Total carnitines; (**B**) Total sphingomyelins; and (**C**): Ceramide enriched with palmitic acid (16:0) in control rats (CT), or rats submitted to MI fed a standard (AMI) or a high fat diet (HFD-AMI). Bar graphs represent the mean±SEM. of 8-10 animals normalized to CT group. *P<0.05 vs control group. † P<0.05 vs AMI group.

**Figure S6. Impact of myocardial infarction on plasma-derived microRNAs from non-obese and obese rats.** Levels of (A): microRNA (mRNA) mRNA 15a-5p; (B): mRNA 7-1-3p; (C): mRNA 29b-3p and (D): miRNA34a-5p in control rats (CT), or rats submitted to MI fed a standard (AMI) or a high fat diet (HFD-AMI).. Bar graphs represent the mean±SEM of 8-10 animals normalized to CT group.

Figures 2C-2F

**Figure S7. Original immunoblots for indicated figures.**

**Table S1.** Raw data of the 11 selected miRNAs from pooled samples.

**Table S2. Association between systolic and diastolic function and myocardial fibrosis, levels of cardiac triglycerides, plasma miRNAs in rats s**ubmitted to myocardial infarction fed a standard diet or a high fat diet and rats fed a control diet and with SHAM operation.

| **Parameter** | **LVEF** | **SF** | **E/A** |
| --- | --- | --- | --- |
|  | r (p) | r (p) | r (p) |
| Fibrosis | -0.57 (0.001) | -0.525 (0.002) | -0.601 (0.001) |
| Mitochondrial TGs | -0.542 (0.025) | -0.613 (0.009) | -0.611 (0.016) |
| miRNA 15b-5p | 0.593 (0.003) | 0.555 (0.006) | 0.425 (0.055) |
| miRNA 194-5p | 0.454 (0.051) | 0.488 (0.034) | 0.412 (0.089) |

TGs: triglycerides; LVEF: left ventricle ejection fraction; SF: shortening fraction; E/A: E-wave, A-wave ratio.

**Table S3. Association between plasma levels of miRNAs and cardiac lipid species in rats s**ubmitted to myocardial infarction fed a standard diet or a high fat diet and rats fed a control diet and with SHAM operation**.**

| **Parameter** | **TGs** | **CL (18:2)_4_** | **CL (22:6)** | **CL (20:4)** |
| --- | --- | --- | --- | --- |
|  | r (p) | r (p) | r (p) | r (p) |
| miRNA 15b-5p | -0.459 (0.085) | **0.752 <0.0001)** | **-0.472 (0.048)** | -0.457 (0.056) |
| miRNA 194-5p | **-0.674 (0.016)** | **0.574 (0.032)** | -0.498 (0.059) | -0.451 (0.091) |
| miRNA 19a-3p | -0.267 (0.318) | 0.239 (0.355) | **-0.632 (0.005)** | **-0.690 (0.002)** |
| miRNA 144-5p | -0.517 (0.086) | 0.372 0.191) | **-0.786 (0.001)** | **-0.803 (<0.0001)** |
| miRNA 301a-3p | -0.359 (0.228) | 0.452 (0.091) | **-0.567 (0.022)** | **-0.558 (0.025)** |

TGs: triglycerides; CL: cardiolipins

**Table S4. Association between plasma levels of miRNAs and adiposity index and HOMA in rats s**ubmitted to myocardial infarction fed a standard diet or a high fat diet and rats fed a control diet and with SHAM operation**.**

| **Parameter** | **Adiposity Index** | **HOMA** |
| --- | --- | --- |
|  | r (p) | r (p) |
| miRNA 19a-3p | **-0.671 (0.003)** | -0.303 (0.160) |
| miRNA 144-5p | **-0.513 (0.07)** | -**0610 ( 0.007)** |
| miRNA 301a-3p | **-0.547 (0.019)** | -0.363 (0.116) |

**Table S5. Association between plasma levels of miRNAs and cardiac mitochondrial levels of** Lysophosphatidylcholine (Lyso-PC) or Lysophosphatidylethanolamine (Lyso-PE)  **in rats s**ubmitted to myocardial infarction fed a standard diet or a high fat diet and rats fed a control diet and with SHAM operation**.**

| **Parameter** | **Lyso-PC** | **Lyso-PE** |
| --- | --- | --- |
|  | r (p) | r (p) |
| miRNA 1260a | 0.744 (0.001) | 0.845 (0<0.001) |
| miRNA let7f-5p | 0.533 (0.033) | 0.535 (0.04) |
